# Antibody Prevalence and Risk Factors Associated with *Rickettsia* spp. in a Pediatric Cohort: SFGR Remains Underdiagnosed and Underreported in El Salvador

**DOI:** 10.3390/pathogens11111241

**Published:** 2022-10-27

**Authors:** Kyndall C. Dye-Braumuller, Marvin Stanley Rodríguez Aquino, Kia Zellars, Hanna Waltz, Madeleine Meyer, Lídia Gual-Gonzalez, Stella C. W. Self, Mufaro Kanyangarara, Melissa S. Nolan

**Affiliations:** 1 Department of Epidemiology and Biostatistics, Arnold School of Public Health, University of South Carolina, Columbia, SC 29208, USA; 2 Centro de Investigación y Desarrollo en Salud, Universidad de El Salvador, San Salvador, El Salvador

**Keywords:** spotted fever group rickettsioses, serology, *Rickettsia*, risk factors, ELISA, El Salvador

## Abstract

Spotted fever group rickettsioses (SFGR) are caused by a group of tick-borne pathogens that are increasing in incidence globally. These diseases are typically underreported and undiagnosed in low- and middle-income countries, and thus, have been classified as neglected bacterial pathogens. Countries with high poverty, low human development index score, and limited health infrastructure—like El Salvador in Central America—lack necessary surveillance for SFGR and other tick-borne pathogens. This paucity of baseline SFGR infection prevalence leaves vulnerable populations at risk of misdiagnosis. Further, tick-borne disease burdens in El Salvador are severely limited. To lay the foundation for tick-borne disease epidemiology in El Salvador, our team conducted two different enzyme-linked immunosorbent assays (ELISA) on banked human sera samples from a cohort of approximately 1000 pediatric participants from a high-risk vector-borne disease population. Eleven percent of all tested banked pediatric sera were positive for at least one ELISA assay at the time of enrollment: 10.7% were positive for only IgM antibodies (acute SFGR infection), and 2.5% were positive for IgG antibodies (a past SFGR infection). Older, male, children enrolled during the wet season, with a household history of infectious disease and higher maternal education level had higher odds of SFGR antibodies. Additionally, children from households with domestic poultry birds and previous knowledge of other vector-borne diseases had significantly reduced odds of SFGR antibodies. The large percentage of acute SFGR infections indicates that it continues to remain an underreported and undiagnosed issue in El Salvador and the Central American region. Much is still unknown regarding the complexity of the tick, animal host, and human host ecology transmission cycle of SFGR in El Salvador.

## 1. Introduction

Spotted fever group rickettsioses (SFGR) are a pathogen group encompassing multiple *Rickettsia* bacteria species transmitted by hard ticks (Acari: Ixodidae). Clinical symptoms typically include rash, fever, headache, myalgia, and in more severe cases, meningoencephalitis, multiorgan failure, or death [1,2,3]. The most common symptomology falls under the large umbrella of undifferentiated febrile illness (UFI). These bacteria have a global distribution but are most common in subtropical or tropical areas. In the United States, where surveillance is fairly comprehensive, SFGR incidence has increased annually [4,5]. In other regions, widespread surveillance is not comprehensive, and estimates of incidence are severely lacking [6]. However, anthropogenic changes including urbanization and climate change are thought to increase SFGR pathogen distribution through vector habitat disturbances leading to increased risk for human disease [7,8]. SFGR as a pathogen family comprises multiple species causing disease along the clinical spectrum. For example, *Rickettsia amblyommatis* is believed to cause mild infection versus *Rickettsia rickettsii* which is known to cause severe morbidity and a variety of clinically relevant species in between [9]. Although easily treatable with antibiotics, if cases are not diagnosed and remain untreated, the likelihood of severe disease increases [10]. In higher-income countries, case fatality rates rarely rise above 10%; however, in low- and middle-income countries, especially in Latin America, case fatality rates have reached between 20–55% [11,12,13,14]. Unfortunately, the common UFI symptoms displayed are shared among multiple neglected tropical pathogens (e.g., arboviruses, soil-transmitted helminths, etc.), and specific diagnostic methods for the tick-borne disease are either unreliable or too costly for countries with constrained public health resources [10,15]. This is the case for El Salvador, where most neglected tropical disease (NTD) public health resources are devoted to mosquito-borne disease treatment and surveillance [11]. Without a baseline understanding of tick-borne disease burden, infected individuals are more likely to remain undiagnosed and the true burden of the disease will be underreported [10].

Two prior *Rickettsia* serosurveys from the early 1990s identified SFGR prevalence ranging from 32.5–40% among small tested groups (n = 40 in each report) [16,17]. However, neither of these reports evaluated reactive antibody type, therefore infection stage or temporal pathogen exposure could not be determined. While not always exclusive in infection-timing determination, this lack of detail in surveillance can place public health officials at a disadvantage when there is little to no infection time sequence. Additionally, neither of the previous studies investigated the epidemiological characteristics of positive individuals. In contrast, four reports from the neighboring country Honduras have identified frequent contact with animals (specifically, dogs) and large amounts of time spent outdoors as significant risk factors associated with SFGR antibody seroprevalence [18,19,20,21]. In Nicaragua, exposure to farm animals and dogs, rural residence, and lower education were all found to significantly increase human SFGR infection odds [22,23,24]. Lastly, a fatal outbreak of SFGR in Guatemala also suggested those in close contact with dogs, rats, and farm animals were at the highest risk for infection [25].

Without this epidemiological information in El Salvador, public health officials are left at an impasse when determining where to dedicate limited resources to break the infectious transmission cycle. To understand the burden of tick-borne rickettsial pathogens and potential risk factors for SFGR infection in a vulnerable population in El Salvador, our team conducted IgM and IgG ELISA antibody serological assays on banked pediatric participant samples and performed an epidemiological risk factor evaluation.

## 2. Materials and Methods

### 2.1. Study Recruitment

Participants recruited for the original study were from the Department of Sonsonate, El Salvador, located in the western region of the country. This region is made up of 16 municipalities, and participants were approached for enrollment from the entire department. Detailed information on the study site and recruitment of pediatric participants for this research are described in the previous study [26]. Briefly, this study prospectively enrolled approximately 1100 children aged 9 months to 18 years from January to December 2018 for a vector-borne disease surveillance cohort that also investigated concomitant intestinal parasitic infection and malnutrition. Enrolled children originated from either (1) acute undifferentiated febrile children recruited from state pediatric clinics, or (2) asymptomatic children from homes known to the state vector control agency to have triatomine infestation. All sera samples have been bio-banked at −80 °C since initial collection. A household survey was conducted, ascertaining household composition, demographics, and poverty indicators by interviewing the legal parent or guardian of enrolled children. Chagas disease, gastrointestinal parasites, and malnutrition status were collected from the prior study for covariate assessment in the current investigation.

### 2.2. Ethics Statement

Institutional Review Board or human ethics committee approvals were secured from the El Salvadoran Ministry of Health, the University of South Carolina, and Baylor College of Medicine. Parental or legal guardian signed consent and verbal child assent were secured through study nurses prior to participation or sample collection. Recruitment, consent, and data collection were all conducted in Spanish by fluent, native Spanish-speaking study personnel who were fully trained in the protection of human research subjects and in interviewing techniques to cater to local cultural appropriateness. All approached participants and guardians were provided a non-monetary (food) appreciation gift for their time and consideration.

### 2.3. Antibody Screening

For this study, commercially available SFGR IgG and IgM antibody ELISA kits were used (Fuller Laboratories, Fullerton, CA, USA). All patient samples were screened at a 1:100 dilution and the manufacturer’s recommended methods and protocols were followed. In short, ELISA testing methods were as follows: patient sera were diluted, incubated for 1 h at ambient temperature (22–25 °C), then washed. Enzyme-labeled goat anti-human IgM or IgG enzyme conjugate was added for the appropriate ELISA, followed by a 30 min incubation period. Lastly, an enzyme substrate was added to each reaction well to allow for any antigen-antibody reactions in a darkened environment. Using the same incubator conditions, the ELISA plate was placed in an aluminum foil-covered box to prevent any light from entering. Reactions were stopped following 10 min reaction time, and the absorbance of each sample was read through a 96-well microplate reader at 450 nm (Accuris Instruments, London, UK).

The IgM antibody kit measures an early immune response (acute or current infection) to SFGR antigens, while the IgG kit measures any immune response (past exposure) to SFGR antigens. The IgM antibody kit utilizes antigens purified from *R. rickettsii* specifically; however, these antigens will react with other closely related SFGR species including *R. parkeri* and/or *R. africae*. The IgG antibody kit utilizes a group-specific lipopolysaccharide antigen extracted from SFGR species, which will react to multiple SFGR species including: *R. rickettsii*, *R. parkeri*, *R. amblyommatis*, *R. conorii*, *R. siberica*, *R. australis*, and/or *R. akari*. A cutoff calibrator was used for discrimination between reactive and non-reactive sera. By dividing the absorbance values of patient sera by the average absorbance values of the cutoff calibrator, an index value was derived, and the cutoff calibrator was set at an index of 1.0. According to the IgG ELISA manual, samples with index values > 1.1 were considered positive, index values 0.9–1.1 were considered equivocal, and index values < 0.9 were considered negative for IgG antibodies. According to the IgM ELISA manual, samples with index values > 1.2 were considered positive, index values 0.8–1.2 were considered equivocal, and index values < 0.8 were considered negative for IgM antibodies.

### 2.4. Data Analysis

SFGR antibody prevalence was determined for both acute or current infection (IgM positives) and historical infection (IgG positives). Chi-square and Fisher’s exact tests were performed to assess potential associations between SFGR infection positives with previously determined Chagas disease infection, gastrointestinal (GI) parasite infection, and febrile status. The total number of Chagas-positive individuals (n = 25) from the entire pediatric cohort was low, and there were relatively few individuals tested for GI parasites; thus, Fisher’s exact tests were necessary to interpret these comparisons.

The participant household survey from the original study included multiple variables that were analyzed as potential risk factors for SFGR antibodies including date of enrollment, demographic characteristics, poverty indicators, knowledge of the vector-borne disease, association with animals, and health information. Descriptive statistics were ascertained on all participants and stratified by the outcome: antibody-positive and antibody-negative individuals. Three separate multivariable logistic regression models with stepwise selection were run to estimate associations between potential risk factors and antibody status. Initial inclusion into each model was set to *p* < 0.35, and final inclusion was set to *p* < 0.10. Three models were run to determine risk factor differences between individuals positive for the two separate SFGR antibodies. Model 1 regressed the outcome of positive/negative on either SFGR ELISA against various risk factors, Model 2 utilized the outcome of positive/negative on IgG ELISA, and Model 3 utilized the outcome of positive/negative on IgM ELISA. Multicollinearity was assessed for all variables included in the stepwise selection models through variance inflation factors; no multicollinearity was found among any variables. All analyses were executed in SAS statistical software (version 14.1; SAS Institute Inc., Cary, NC, USA).

## 3. Results

**Figure 1:**
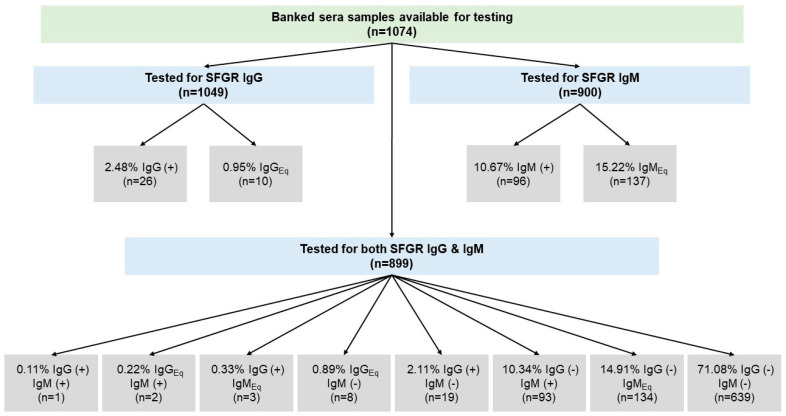
Breakdown: Prevalence of IgG and IgM serology results. Abbreviations/Symbols are defined as: Eq = equivocal, (−) = negative, and (+) = positive ELISA result.

A total of 1175 eligible participants were approached for enrollment, and 1074 participants consented, completed interviews, provided serum samples, and were included in the original study’s final sample [26]. Of the available 1074 participants’ banked serum samples, a total of 1049 (97.7%) were tested for either IgG or IgM antibodies or both (Figure 1). Twenty-five participants’ sera were insufficient volumes for any test, and IgG diagnostic was prioritized for the 149 participants’ sera whose volume only permitted one test. In summary, 1049 samples were tested for IgG antibodies, and 900 samples were tested for IgM antibodies. A total of 899 participants’ samples were tested for both antibodies.

From the banked sera tested for IgG antibodies, 2.48% (n = 26) and 0.95% (n = 10) tested positive and equivocal, respectively, indicating a past convalescent infection to SFGR species. From the sera tested for IgM antibodies, 10.67% (n = 96) and 15.22% (n = 137) tested positive and equivocal, respectively, indicating a current or acute infection with SFGR bacteria. Figure 1 includes additional information on the breakdown of sera samples positive and equivocal for multiple assays or those positive or equivocal for at least one assay. Approximately 11% of all tested samples were positive for at least one ELISA assay, and 25% of samples tested either positive or equivocal on at least one ELISA assay. A total of 5 (0.56%) participants’ sera samples were positive for one ELISA and equivocal for another. Only one participant tested positive on both IgG and IgM assays (0.11%). Of those tested for both antibodies, 14.91% (n = 134) tested negative for IgG antibodies and equivocal for IgM antibodies. Additionally, 0.89% (n = 8) tested negative for IgM antibodies and equivocal for IgG antibodies.

**Figure 2:**
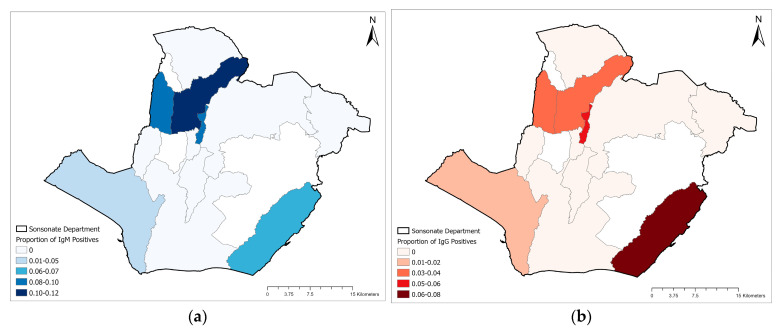
Geographic distribution of serology proportion results from ELISA assays: (**a**) IgM-positive results only; (**b**) IgG-positive results only.

The geographic distribution of ELISA antibody results in the Sonsonate Department is displayed in Figure 2. The proportion of IgM positives is displayed in Figure 2a by municipality, and IgG positives are displayed in Figure 2b. The distribution is similar between both antibody distribution maps. The highest concentration of IgM seropositive individuals appears to be in the Nahuizalco municipality, in the north-central region. The neighboring municipalities Santa Catarina Masahuat directly to the west, and Sonzacate to the southeast have the second highest proportions of IgM-positive individuals, followed by Santa Isabel Ishuatán municipality in the southeastern corner and Acajutla municipality in the southwest corner. This pattern is similar for the proportion of IgG seropositive individuals, except the Santa Isabel Ishuatán municipality has the largest proportion of IgG-positive individuals.

**Figure 3:**
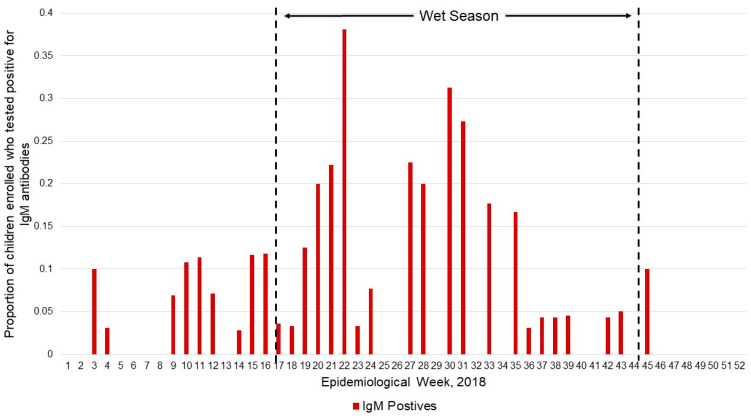
Seasonality of SFGR serology in enrolled pediatric participants, proportion positive of all enrolled.

During 2018, the majority of enrolled IgM-positive participants were enrolled during the wet season, from Epidemiological Weeks (EpiWeek) 18–44 (Figure 3). There was a steady percentage of participants testing positive for IgM antibodies (around 10% of participants) weekly from EpiWeek 3 to EpiWeek 19, with a dramatic peak at almost 40% of participants at EpiWeek 22. This percentage of IgM positives dropped to less than 5% of participants at EpiWeek 36; the last positive participants were enrolled in EpiWeek 45.

There were no associations at the 0.05 significance level between serology results and previous Chagas disease infection, GI parasite infection, or febrile status (Table S1). Potential associations of interest (*p*-value < 0.10) included those between IgG positive status and Chagas disease status, either ELISA positive status and Chagas disease status, and either ELISA positive status and febrile status.

Household and individual demographic and risk factor information from the original study enrollment survey is provided in Table 1 [27]. Results are broken down into three SFGR antibody status groups: positive/negative on at least one ELISA, positive/negative on IgG ELISA only, and positive/negative on IgM ELISA only. Equivocal test results were excluded. Multivariable results are presented in the next paragraph, but here we present the overall cohort’s descriptive statistics. The majority of antibody-positive individuals were male, not febrile, enrolled during the wet season, and were slightly older than those who were antibody negative; however, a male gender association was not noted among IgG positives. Household occupancy and composition were also very similar among groups, with slightly more individuals living in antibody-negative participants’ homes and IgG antibody-positive individuals with the lowest average last year of maternal education, comparatively. Poverty indicators appeared to be evenly split between antibody-positive and negative individuals, except a larger proportion of positive individuals had adobe walls in their bedrooms, potable water, and no electricity, while a smaller proportion of positive individuals consistently reported having fowl (chickens, ducks, and/or turkeys). Additionally, a smaller proportion of antibody positives consistently reported higher vector-borne disease knowledge, assessed through the proxy variables knowing what a “chinche” is, someone in the household being bitten by a chinche, and having the house treated/fumigated in the past year, compared to antibody negative individuals. A “chinche” is a local Spanish term for triatomine, the vector for Chagas disease parasites [26]. Lastly, more antibody-positive participants reported someone in their household needing medical attention for an infectious disease in the past year than antibody-negative participants.

Multiple risk factors were significantly associated with serology status in the pediatric cohort, also included in Table 1. One variable was significantly associated with anti-body positive status in all three models: participant age. As age increased by one year, participants’ odds of testing positive for either SFGR antibody (Model 1) significantly increased (*p*-value < 0.0001; OR: 1.16, 95% CI: 1.08 to 1.23). A similar increase in odds was seen in the model for IgG ELISA positive only (*p*-value = 0.0159; OR: 1.18, 95% CI: 1.03 to 1.35) and IgM positive only participants (*p*-value < 0.0001; OR: 1.15, 95% CI: 1.08 to 1.24).

The adjusted odds of testing *Rickettsia* antibody positive among males was between 1.8 and 5.3 times as high as females in the first (*p*-value = 0.0456; OR: 1.81, 95% CI: 1.01, 3.25) and second models (*p*-value = 0.0203; OR: 5.31, 95% CI: 1.40, 20.25). As mother’s last year of education increased, Model 1 and Model 3 indicated participants’ adjusted odds of testing positive for SFGR antibodies increased (Model 1 *p*-value = 0.0738; OR: 1.07, 95% CI: 0.99 to 1.15; Model 3 *p*-value = 0.0115; OR: 1.11, 95% CI: 1.02 to 1.21). Although Model 1’s OR is not significant, this trend is of note. A household with reported member(s) seeking clinical care for an infection origin in the past year had 2.17 times the adjusted odds (*p*-value = 0.0094; 95% CI: 1.21 to 3.91) of antibody-positive status for either ELISA, and the same pattern was seen for the odds of IgM antibody positive participants only (*p*-value = 0.0516; OR: 1.90, 95% CI: 0.99 to 3.62)—although this was only slightly higher than a 0.05 significance level. Both Models 1 and 3 also indicated that participants who were febrile at enrollment had a decreased adjusted odds of either antibody positivity Model 1 *p*-value = 0.0035; OR: 0.36, 95% CI: 0.18 to 0.71) and IgM antibody positivity only (Model 3 *p*-value = 0.0132; OR: 0.38, 95% CI: 0.17 to 0.82). The adjusted odds of antibody positive status on either ELISA assay for those who indicated they used an outside latrine for a bathroom were 0.249 times the odds of those who did not use this type of bathroom (*p*-value = 0.0011; OR: 0.25, 95% CI: 0.11 to 0.58). This same pattern was seen in IgG positive participants (*p*-value = 0.0027; OR: 0.10, 95% CI: 0.03 to 0.42).

For Model 1 only, those who indicated they did not have electricity had 2.4 times the adjusted odds (*p*-value = 0.0048; OR: 2.41, 95% CI: 1.31 to 4.45) of SFGR antibody-positive status compared to those who did have electricity. Households with birds had a decreased adjusted odds (*p*-value = 0.0156; OR: 0.49, 95% CI: 0.27 to 0.87) of testing positive for either antibody, and those reporting someone in the household bitten by chinches in the past year had a decreased adjusted odds (*p*-value = 0.0491; OR: 0.39, 95% CI: 0.16 to 0.99) of testing positive for either antibody.

For Model 2 only, households with a cement bedroom floor had decreased adjusted odds of SFGR IgG antibodies (*p*-value = 0.0276, OR: 0.26, 95% CI: 0.08 to 0.86) compared to those with a bare earth bedroom floor. Additionally, those reporting that they knew what a chinche was had decreased adjusted odds for IgG antibodies (*p*-value = 0.0027, OR: 0.13, 95% CI: 0.03 to 0.49) compared to those who did not know what a chinche was.

For Model 3 only, participants enrolled during the wet season had 2.5 times the odds of testing positive for SFGR IgM antibodies compared to those enrolled during the dry season (*p*-value = 0.0099; OR: 2.53, 95% CI: 1.25, 5.11).

## 4. Discussion

This is the first report in over 25 years investigating the serological evidence of SFGR infection and potential SFGR risk factors in El Salvador, adding necessary and updated information to the literature regarding human infection with the rickettsial disease in Central America. Our results indicate a smaller potential burden of past or current infections with SFGR pathogens than previously reported. In 1987, the World Health Organization (WHO) developed an IFA assay for the detection of antibodies to multiple rickettsial pathogens and distributed this with protocols to laboratories in 37 countries, and one laboratory in El Salvador participated in this effort. From 40 human sera samples tested, 13 (32.5%) tested positive for SFGR antibodies, but the antibody type was not described [17]. A second study also surveyed 40 human sera samples from El Salvador and found 16 (40.0%) positive with the presence of SFGR antibodies [16]. A description of these previous studies’ population demographics (pediatric vs. adult, febrile vs. afebrile, etc.) is not provided, so our results are not directly comparable. However, our study does provide a baseline understanding of past and acute SFGR infections for children in Sonsonate less than 18 years old. Because IgM antibodies typically peak following 3–4 weeks post-infection—and are detectable for 3–4 months—we believe IgM-positive participants were infected with SFGR bacteria within a 3–4 month window of enrollment [27,28,29,30]. IgG antibodies peak 10–12 weeks post-infection and are detectable for up to 1 year, therefore IgG positive participants are likely infected in a much larger window [27,28,29,30]. Our results indicate a small proportion of pediatric participants with a past (1 calendar year) infection through IgG antibody detection (2.48% positive and 0.95% equivocal), but a surprisingly high number of acute or current infections through IgM antibody detection (10.67% positive and 15.22% equivocal). The age of participants ranged from 9 months to 18 years, thus in our study population, a relatively high proportion of children were acutely SFGR infected.

Overall, this is the first report to highlight the proportion of pediatric, El Salvadorian, acute infections, a known vulnerable group for SFGR infections and severe disease [31,32,33,34]. Although our study only includes information from one department, this is crucial information for public health in this country providing a semblance of baseline information for additional areas in the country. Serologic determination of rickettsial infections has been utilized since its inception in the late 1910s when the Weil-Felix (WF) test was developed [35]. Multiple techniques have been developed since; antibodies to *Rickettsia* species can be detected using complement fixation [36], latex agglutination [37], enzyme-linked immunosorbent assay (ELISA) [38], and immunofluorescent assay (IFA) [39]. Both the ELISA and IFA assays have proven to be adequate antibody screening tools for low- and middle-income countries, although, like El Salvador, these tests are rarely implemented in routine pathogen surveillance. While the current study is limited to one department, we hope the built infrastructure from this study will support expanded SFGR diagnostic surveillance throughout the country and region.

The geographic distribution of SFGR antibody positives generally reflected the distribution of parasitic infections earlier reported from this department [26]. The municipalities in northcentral Sonsonate had the highest proportion of antibody-positive individuals, and with this information, public health professionals can potentially begin to narrow down areas in the country where tick-borne disease investigations can be initiated, or risk factor analyses can be attempted. These municipalities lie in a biodiverse forested region at a higher elevation than the surrounding towns, and this ecological habitat is likely supportive of ticks and other vectors. Poverty indicators were generally not associated with seropositivity, and similar vary little geospatially across the department of Sonsonate. Therefore, providing further supporting evidence that the ecological habitat in the northcentral municipalities is likely the driving force for SFGR transmission.

Seasonality played a statistically significant role in the accumulation of IgM-positive individuals, with a peak in seroprevalence rate by weekly enrollment noted within the first several weeks following the transition between the dry and wet seasons. While the kinetics of antibody responses will vary by person, and by the species of SFGR bacteria infecting the individual [29,30], the increased rate of IgM seropositive cases at the wet season inception is likely due to El Salvador’s hotter and more humid climate, providing a preferable habitat for tick vectors’ movement and reproduction. Further, domestic livestock and animals are brought closer to the house or into the house for monitoring during the wet season, increasing human–animal-vector contact exposure. This cultural practice combined with more ticks in the environment questing for blood meals yields an increased chance for children to come into contact with ticks and bacteria transmission. Additionally supporting evidence for seasonal influence is noted by a previously fatal SFGR outbreak in neighboring Guatemala—in a department on the El Salvador border—at the beginning of the wet season in 2007 [25].

Human antibody presence to SFGR rickettsial pathogens has been documented in neighboring countries Guatemala and Honduras, along with nearby countries Belize and Nicaragua. Our results fit within the range of neighboring countries’ reports of SFGR antibodies: one study investigating an outbreak in 2007 reported 40% of tested individuals with antibodies to SFGR [25], and two others reporting serological surveys from Honduras reported individuals with antibodies to SFGR from 0.3–0.9% [40] to 3.9% [20]. In Belize, one study reported human antibody prevalence at 33.5% [41], and studies in Nicaragua have reported SFGR antibody presence in 0.3% [40] to 6.8% [22] of individuals tested. The use of ELISA versus IFA assays was evenly distributed throughout these reports, suggesting our use of the ELISA diagnostic platform has local species relevance. These previous studies further support our suggestion that human infection with tick-borne rickettsial pathogens is probably more common than previously thought, and constant throughout the tropical Central American region.

All three multivariate logistic models identified a number of risk or protective factors associated with SFGR antibody presence in this pediatric cohort. Increased age was the only variable consistently seen to increase participants’ odds for SFGR antibody presence for all three models by approximately 15–18%, suggesting older age-activity-related epidemiologic exposures. A similar age-dependent increase has been documented in two separate studies in Peru, where older participants (older children and adults) had consistently greater odds of SFGR antibodies [42,43]. Both Models 1 and 2 also indicated that males had increased odds for SFGR antibodies, which has also been reported in previous studies in South America [43,44,45]. The World Bank reports a 91% primary school completion rate, yet a notable decline in secondary education from 636,536 in 2013 to 457,610 in 2021 for El Salvador [46]. It is possible that older children sought employment after primary school education completion, thus making them at higher risk for SFGR transmission. Additionally, as children age and grow, their propensity to explore outdoors increases, increasing chances of encountering ticks in the environment—especially from infested dogs [47], and males in general typically participate in activities associated with higher tick exposure due to gender roles and playtime [43,44,48,49,50].

Family use of an outdoor latrine was a significant protective factor against either SFGR antibody or IgG antibodies. This rural poverty indicator is a surprising finding, as poverty indicators are commonly found associated with increased odds of neglected tropical diseases. In previous vector-borne disease studies, outdoor latrine use has been associated with increased odds of visceral leishmaniasis [51], but decreased odds of lymphatic filariasis [52]. One study in Mexico found an outside latrine was associated with decreased risk for SFGR-infected animals around households but did not offer an explanation as to why this could be the case [53]. It is possible outdoor latrines are avoided by feral animals hosting ticks, and this could reduce potential infected tick exposure around these households. Additional factors related to the ecology of SFGR transmission between ticks and host animals potentially related to outdoor latrine use need to be explored.

Another variable with an unanticipated impact on the odds of SFGR antibodies was increasing maternal educational level, which had a positive impact on the odds of at least one ELISA-positive and IgM antibodies (Models 1 and 3). While a statistical association was identified, it should be noted that the maternal education levels modestly differed by serology group, and the overall average maternal education level was low (a primary school education—5th grade). Few studies have included education level in epidemiological investigations regarding SFGR serology, and results either suggest no association between education level and positive serology [54] or an increase in odds for SFGR antibodies with lower education level [22,55,56]. However, these reports reference the respondents’ own level of education and not maternal education level. It is possible that mothers with a higher education level have jobs outside of the home, meaning they might leave their children at home more often than those at home. This could lead to children potentially encountering ticks from infested animals or from the environment more as they are unsupervised more often than others. More recent serological investigations in Latin America focus on parental occupation rather than education level [42,43]. One study found occupations outside of the home (agriculture or non-agriculture based) had elevated odds of SFGR serostatus compared to those who were students or at home [42].

For both Models 1 and 3, participants with a fever (temperature > 38 °C) at the time of enrollment, had a significant reduction in SFGR serology odds compared to those without a fever. We had anticipated that those with potential evidence of an acute infection (IgM positive) would be likely to present with a fever, but this was not observed in the data at the 0.05 significance level [13]. A few explanations exist. First, most acutely infected participants were outside of the febrile clinical window, as this typically develops within 1–2 weeks of infection and lasts for approximately 1 week [57], and IgM antibodies peek around 3–4 weeks—thus this symptom can be missed if serology evaluation is not timed just right. Second, a larger proportion than anticipated of those acutely infected never developed a fever. A growing body of evidence has suggested that many SFGR infections’ symptomologies will not always include fever, and this has been seen in multiple cases [58,59,60]. Given the large differences in symptoms from the various SFGR species, it is important to note that multiple studies have indicated that relying on the most common symptoms of SFGR infections or strict case definitions will miss many infections where symptomology is not ‘typical’ [30,59]. Third, it might be possible that a non-pathogenic SFGR species elicited a humoral immunologic response. While the IgM ELISA targeted *R. rickettsii*, cross-reactivity with other SFGR *Rickettsia* species is possible. Lastly, the recruitment of this population (from the original study) indicated that participants were either presenting at a clinic or known to live in a home infested with triatomines. A selection bias could have occurred here where this selection mechanism could be related to the febrile status, and this was not indicated through our statistical models.

Odds for SFGR serostatus increased significantly when respondents indicated someone in the household had sought clinical care for an infection origin in the past year. This could indicate that residents of the same household are at increased risk for multiple vector-borne diseases due to similar characteristics such as poverty indicators, as seen in the previous Chagas disease study [26]. Additionally, illness of an infectious origin could be relatively common in these households, leading to a potential complacency regarding healthcare access for mild clinical disease. However, we identified a lack of parasitic infection associations. It is important to note that only 25 children were identified as Chagas disease positive from this previous study, and only 168 individuals were screened for GI parasites, thus limiting the number of participants whose SFGR results could be compared.

Our study also indicates that households having domestic fowl such as chickens, ducks, or turkeys had significantly lower odds of either SFGR antibody. Pet bird (macaws and parakeets) ownership was found to be a significant protective factor for SFGR antibodies in one Peru study [42], although our study is the first to assess domestic fowl’s impact on SFGR seropositivity. The presence of birds may serve as a zooprophylactic protective factor to reduce the contact between infected ticks and humans, and it is also postulated that the non-pet species mentioned specifically in El Salvador could directly protect household members through tick predation [61,62].

The prior investigation’s Chagas disease knowledge questions were evaluated as a proxy for vector-borne disease risk in the home and homeowner vector-borne disease knowledge. Participants reporting “someone had been bitten by a chinche in the past year” and those reporting “knowing what a chinche is” had significantly reduced odds for SFGR anti-body status in Model 1 and Model 2, respectively. It is plausible that individuals in households with previous non-tick vector experience (e.g., triatomines) will be more observant or will notice other vectors, i.e., ticks, inside their homes or on family members. This would lead to a reduction in either the number of ticks brought into one’s household or the amount of time ticks could spend on children or family members inside, as more vigilant parents are more aware of arthropod vectors and will remove them.

In contrast, this study investigated two positively associated risk factors for pediatric SFGR. Households without electricity had significantly increased odds of either SFGR antibody type. This poverty indicator is probably related to the amount of time children are spending outdoors: increased outdoor playtime is anticipated when there is no electricity in the household, increasing the chances of encountering infested animals or infected ticks in the environment. Previous studies have seen an increased likelihood of SFGR infection and other vector-borne diseases within households without electricity [63,64]. Another poverty indicator, the type of floor in the bedrooms, was a significant predictor for IgG antibodies in participants. Cement floors, as opposed to bare earth floors, significantly reduced the odds of SFGR antibody serology. Dirt floors are common in more rural areas, which is a typical risk factor for SFGR infection where greater human–animal-environmental interactions occur [64].

A few non-statistical model results were surprising: whether or not the household had dogs and whether the primary household income source was agriculture-based. Exposure to dogs is one of the most commonly reported risk factors for SFGR infection in the Central American region [18,22,23,24,25]. Although, dog ownership was not found to be significantly associated with increased odds for SFGR antibodies in some studies in South America [42]. Dogs, and canines in general, are thought to be important bridges from the sylvatic environment to the domestic, great hosts for multiple species of ticks, and they can develop sufficient bacteremia to sustain transmission of SFGR bacteria for 4–8 days, on average, post-infection [65]. In addition to dog exposure, previous reports have indicated copious amounts of time spent outdoors [18,20], agricultural work [22,25], and exposure to other animals like rodents [19,21,25] are all significant risk factors for SFGR disease infection. The original study of this pediatric cohort did find dogs were a significant risk factor for Chagas disease infection [26], but it seems as though households reporting dog ownership were spread relatively evenly among the SFGR antibody positive and negative groups. Additional information regarding animals in and around the home would be beneficial to further understand this ecological dynamic.

Limitations to this study include the fact that SFGR serological assays are only diagnostically confirmative when paired with acute and convalescent samples, and the cross-sectional nature of this study design inhibited formal diagnosis. Additionally, both commercially available IgG and IgM ELISAs used in this study do not have the ability to differentiate between rickettsial pathogens and will indicate reactions for antibodies to multiple species of *Rickettsia*. It is thus impossible to determine what species of SFGR individuals may have been infected with without confirmatory molecular testing through polymerase chain reaction (PCR) testing for targeted *Rickettsia* species in patient blood samples Even more challenging is the fact that molecular diagnostics to detect pathogen DNA in samples needs to be conducted at the right time when bacteria are circulating in patient blood. Diagnostics for SFGR infections need multiple upgrades in sensitivity and specificity. The original study and questionnaire were developed to investigate Chagas disease risk factors and were not specifically made for investigating SFGR risk factors or symptomology. Symptomology could have been used to narrow down *Rickettsia* spp. or yielded additional clinical correlate information. The large number of febrile participants also makes comparisons to the rest of the country less generalizable, and the recruiting mechanism targeted those in clinics or living in triatomine-infested homes. Lastly, because this cohort was limited to one department in El Salvador, these results may not be directly comparable to the rest of the country.

The results of this study are important for neglected tropical disease awareness in the Central American region. We demonstrate a relatively high proportion of children with evidence of acute infection or developing seroconversion with SFGR pathogens in El Salvador. Based on these results, we can suggest that there is a large proportion of vulnerable individuals that are at risk for SFGR infection in this country that are potentially remaining undiagnosed. The few serological investigations for El Salvador emphasized the same claim: that SFGR infection in this region is most likely underreported and undiagnosed. Rickettsial antibodies in the body decline over a year [66], and thus the window for identifying infected individuals wanes over time, leaving these individuals with a smaller chance of receiving treatment. Certain rickettsial species, if undiagnosed, can result in severe cases of the disease, sometimes leading to death [1,2,11,12]. This risk is elevated in children, marginalized populations, women, and those in poverty [31]. Diagnostic methods for SFGR infection are not ideal, and specifically in this region, the rickettsial disease presents with typical undifferentiated febrile illness, making these infections notoriously difficult to diagnose [1,10,15]. Additional difficulties arise from the fact that most neglected tropical disease surveillance and treatment in this region is targeted toward other arthropod-borne pathogens including dengue, malaria, Chagas disease, and leishmaniasis [11,22,67].

We found multiple characteristics that increased participants’ odds of SFGR anti-body positivity status, some of which do not fit the typical mold of tick-borne disease ecology and need to be explored even further. The evidence of relevant human behavior and characteristics related to poverty and tick ecology indicates that there are avenues where epidemiological intervention can succeed in preventing future SFGR infections in those at greatest risk in El Salvador. Although singular antibody assay results are not 100% reliable, we present evidence that SFGR infection is likely endemic in Western El Salvador.

Further studies should investigate the burden of rickettsial pathogens in humans, animals, and ticks through formal molecular diagnostic methods. Patients presenting with undifferentiated febrile illness or fever of unknown origin with any of these presented risk factors should be screened for rickettsial pathogens in El Salvador to ensure that SFGR misdiagnosis does not continue in this country and that those who need antibiotics are treated. With this information, we hope that public health officials and infectious disease physicians consider rickettsial infections as a cause of disease to increase tick-borne pathogen awareness and the number of individuals receiving treatment.

## Figures and Tables

**Table 1:** Descriptive Statistics and Risk Factor Information of Enrolled Pediatric Participants, by Antibody Status.

	ELISA IgG and/or IgM			ELISA IgG			ELISA IgM		
	Positive N (%) or Mean ± SD	Negative N (%) or Mean ± SD	Model 1 Odds Ratio (95% CI) ꜝ	Positive N (%) or Mean ± SD	Negative N (%) or Mean ± SD	Model 2 Odds Ratio (95% CI)	Positive N (%) or Mean ± SD	Negative N (%) or Mean ± SD	Model 3 Odds Ratio (95% CI)
	N = 121	N = 887		N = 26	N = 971		N = 95	N = 662	
**Participant characteristics**									
Male	58 (47.9)	478 (53.9)	1.81 (1.01, 3.25) ^†^	17 (65.4)	442 (45.5)	5.33 (1.40, 20.25) ^†^	41 (43.2)	328 (48.0)	
Febrile at time of enrollment	32/117 (27.4)	540 (64.2)	0.36 (0.18, 0.71) ^‡^	7 (26.2)	322/922 (34.9)		25/91 (27.5)	228/642 (35.5)	0.38 (0.17, 0.82) ^†^
Enrolled in the wet season	81 (66.9)	503 (56.7)		14/26 (53.8)	561 (57.8)		67 (70.5)	380 (57.4)	2.527 (1.25, 5.11) ^‡^
Age (years)	10.6 ± 4.6	8.5 ± 4.7	1.16 (1.08, 1.23) *	12.0 ± 4.1	8.6 ± 4.7	1.18 (1.03, 1.35) ^†^	10.2 ± 4.7	8.3 ± 4.8	1.15 (1.08, 1.24) *
**Household occupancy and composition**									
N living in household	5.2 ± 2.1	5.5 ± 3.0		5.0 ± 2.0	5.5 ± 3.0		5.3 ± 2.1	5.5 ± 3.1	
N kids in household	2.5 ± 1.3	2.5 ± 1.4		2.4 ± 1.4	2.5 ± 1.4		2.6 ± 1.3	2.5 ± 1.3	
Mother’s last year of education	5.1 ± 4.0	5.4 ± 4.1	1.07 (0.99, 1.15)	4.2 ± 4.6	5.4 ± 4.1		5.5 ± 4.0	5.4 ± 4.0	1.11 (1.02, 1.21) ^†^
Father’s last year of education	5.5 ± 4.2	6.0 ± 4.4		5.0 ± 4.5	5.9 ± 4.4		5.7 ± 4.1	6.1 ± 4.4	
N beds in home	4.7 ± 1.9	4.8 ± 2.3		4.7 ± 1.9	4.8 ± 2.2		4.7 ± 1.9	4.8 ± 2.3	
**Household poverty indicators**									
Agriculture as primary household income source	69/110 (62.7)	513/816 (62.9)		13/22 (59.1)	564/894 (63.1)		57/88 (64.8)	389/607 (64.1)	
Type of cooking fuel used									
Firewood	14/119 (11.8)	126/886 (14.2)		1/25 (4.0)	139/970 (14.3)		13 (13.7)	95/660 (14.4)	
Gas	6/119 (5.0)	50/886 (5.6)		1/25 (4.0)	53/970 (5.5)		5 (5.3)	37/660 (5.6)	
Both	99/119 (83.2)	710/886 (80.1)		23/25 (92.0)	778/970 (80.2)		77 (81.0)	248/660 (37.6)	
Type of floor in bedroom									
Bare earth	96/119 (80.7)	695 (78.4)		18/25 (72.0)	765 (78.8)		79 (83.2)	514/661 (77.8)	
Cement	23/119 (19.3)	192 (21.7)		7/25 (28.0)	206 (21.2)	0.26 (0.08, 0.86) ^†^	16 (16.8)	147/661 (22.2)	
Type of wall material in bedroom									
Blocks	21/119 (17.6)	177/885 (20.0)		2/25 (8.0)	191/969 (19.7)		19 (20.0)	132/559 (20.0)	
Adobe	58/119 (48.7)	357/885 (40.3)		12/25 (48.0)	401/969 (41.4)		46 (48.4)	279/559 (42.3)	
Other	40/119 (33.6)	351/885 (39.7)		11/25 (44.0)	377/969 (38.9)		30 (31.6)	248/559 (37.6)	
Family uses outside latrine for bathroom	108/120 (90.0)	823/886 (92.9)	0.25 (0.11, 0.56) ^‡^	21/25 (84.0)	901/970 (92.9)	0.10 (0.03, 0.42) ^‡^	88 (92.6)	618/660 (93.6)	
Potable water in the house	112/120 (93.3)	776 (87.5)		24/25 (96.0)	854 (88.0)		89 (93.7)	581/660 (87.9)	
No electricity	58/116 (50.0)	308/876 (35.2)	2.41 (1.31, 4.45) ^‡^	12/24 (50.0)	351/957 (36.7)		46/92 (50.0)	233/651 (35.8)	
House has fowl (chickens, turkeys, and/or ducks)	53/113 (46.9)	471/860 (54.8)	0.49 (0.27, 0.87) ^†^	9/25 (36.0)	511/937 (54.5)		44/88 (50.0)	336/636 (52.8)	
House has cats	17/113 (15.0)	121/860 (14.1)		2/25 (8.0)	135/937 (14.4)		15/88 (15.8)	88/636 (13.3)	
House has dogs	60/113 (53.1)	462/860 (53.7)		11/25 (44.0)	507/937 (54.1)		50/88 (56.8)	335/636 (52.7)	
**Proxy assessment of vector-borne disease exposure and knowledge: household Chagas disease vector exposure and parent or legal guardian Chagas disease knowledge**									
Knows what a “chinche” is	74/120 (61.7)	610 (68.8)		13/25 (52.0)	663 (68.3)	0.13 (0.03, 0.49) ^‡^	61 (64.2)	449/661 (67.9)	
Knows about Chagas disease	17/120 (14.2)	178 (20.1)		6/25 (24.0)	188 (19.4)		11 (11.6)	126/661 (19.1)	
Has seen chinches inside house within the past year	28/120 (23.3)	283 (31.9)		6/25 (24.0)	302 (31.1)		22 (23.2)	218/661 (33.0)	
Someone in household has been bitten by a chinche in the past year	18/120 (15.0)	161/884 (18.2)	0.39 (0.16, 0.99) ^†^	3/25 (12.0)	176/968 (18.2)		15 (15.8)	132/660 (20.0)	
House has been fumigated in the past year	7/120 (5.8)	90/885 (10.2)		2/25 (8.0)	94/969 (9.7)		5 (5.3)	66/660 (10.0)	
**Family health concerns**									
Someone in the household sought clinical care in the past year	90/119 (16.0)	599/867 (69.1)		18/25 (72.0)	665/950 (70.0)		73/94 (77.7)	455/646 (70.4)	
Someone in the household sought clinical care for an infection in the past year	59/118 (50.0)	341/865 (39.4)	2.17 (1.21, 3.91) ^‡^	12/25 (48.0)	386/948 (40.7)		48/93 (51.6)	257/645 (39.8)	1.90 (0.99, 3.62)

ꜝ Variable significant on multivariate regression are as follows: ^†^ *p*-value < 0.05; ^‡^ *p*-value < 0.01; * *p*-value < 0.001. The denominator for all variables is the total N listed in the appropriate column for each group, unless otherwise stated with a different denominator due to missing data.

## Data Availability

Data presented in this study are available upon request from the corresponding author.

## Supplementary Materials

The following supporting information can be downloaded at: https://www.mdpi.com/article/10.3390/pathogens11111241/s1, Table S1: Chi-Square Test of Independence on SFGR ELISA Serology and Parasite Infections.
