# Supplementary material for: Antibody Prevalence and Risk Factors Associated with Rickettsia spp. in a Pediatric Cohort: SFGR Remains Underdiagnosed and Underreported in El Salvador
Source: Pathogens. 2022 Oct 27;11(11):1241. doi: 10.3390/pathogens11111241 (PMC9696242; doi:10.3390/pathogens11111241)
Supplement: Supplementary file 1 [file pathogens-11-01241-s001.zip › pathogens-1966708-supplementary.pdf]

Supplementary Files

Table S1. Chi-Square Test of Independence on SFGR ELISA Serology and Parasite Infections.

	X^2 Test Statistic	X^2 *p*-value	Fisher' Exact Test Probability
Positive for at least one ELISA (IgG or IgM)			
Chagas disease positive [1]	3.48	--	0.0744
Any GI parasite positive [1]	0.0008	--	1.0000
Febrile	3.02	0.082	0.0959
IgG Positive			
Chagas disease positive [1]	5.15	--	0.0785
Any GI parasite positive [1]	0.74	--	0.4265
Febrile	0.71	0.398	0.5316
IgM Positive			
Chagas disease positive [1]	1.53	--	0.1981
Any GI parasite positive [1]	0.21	--	1.00
Febrile	2.28	0.131	0.1572

[1] At least one cell had expected counts less than 5; thus, Fisher's exact test probability is provided.
